# Tuberculosis in schools: an outbreak in northeastern Italy and some key health protection interventions

**DOI:** 10.3325/cmj.2021.62.90

**Published:** 2021-02

**Authors:** Luca Cegolon, Giuseppe Mastrangelo, Davide Gentili, Mario Mastromarino, Andrea Cegolon, Giuseppe Pichierri, Sandro Cinquetti, Saverio Bellizzi, Giovanni Sotgiu

**Affiliations:** 1 Local Health Unit N.2 "Marca Trevigiana", Public Health Department, Treviso, Italy l.cegolon@gmail.com; 2 Department of Cardiac, Thoracic, and Vascular Sciences & Public Health, Padua University, Padua, Italy; 3 Department of Medical, Surgical and Experimental Sciences, University of Sassari, Sassari, Italy; 4 Department of Political, Social and International Relationships, University of Macerata, Macerata, Italy; 5 Department of Microbiology, Kingston Hospital NHS Foundation Trust, Kingston upon Thames, United Kingdom; 6 Partnership for Maternal, Newborn & Child Health, Geneva, Switzerland

## The outbreak

In April 2019, a tuberculosis (TB) outbreak, likely triggered by re-activation of a highly contagious latent tuberculosis infection (LTBI) in a school teacher, was reported in a primary school in the Veneto Region (northeastern Italy). The infection, having probably been dormant for decades, rapidly spread further to two school teachers and up to 11 pupils, a total of 13 cases of active TB that received appropriate TB treatment. Ten out of the remaining 95 school staff members (10.5%) and 34/546 (6.2%) remaining school pupils tested positive on tuberculin skin test (TST). Moreover, 2% (2/97) of pupils attending the first year of junior secondary school within the same municipality during 2017/18 (having completed the above primary school) were also found positive on TST. Since none of 3 school teachers or 84 pupils who completed the last year of primary school in 2017 and attended the second year of a junior secondary school were found to be TST positive, the beginning of the outbreak was dated from January 2018 onwards. Therefore, active TB in the index case may have been potentially infectious and undiagnosed for about 14 months, leading to extensive exposure of close contacts, especially students (1). Anyone with recognized LTBI status – among school staff or pupils – was placed on prophylaxis with isoniazid for 6 months (1).

## Tuberculosis surveillance in schools: health protection laws and policies

TB is a global health priority for the World Health Organization, since it has caused 1.5 million deaths in 2018 and roughly a quarter of the adult world's population is estimated to carry LTBI (2). In the European Union (EU) and the European Economic Area (EEA), TB is still a public health concern (3). While most EU/EEA countries, including Italy, nowadays show a low incidence rate (<10 per 100 000), 55 337 TB cases were still recorded during 2017 in the entire EU/EEA at a rate of 10.7 per 100 000 (3).

One of the key interventions to meet the United Nations Sustainable Development Goal of stopping the global TB epidemic by 2030 (4) is tackling LTBI by providing preventive treatment to persons at high risk of developing infectious TB (5). More than a third of people living with TB are neither diagnosed, treated, nor even notified, which represents an enormous hurdle for the disease control and elimination (5,6). Moreover, migration from high-risk areas has changed the pattern distribution of TB in high income countries over the last few decades (7). The proportion of foreign-born TB cases in the EU/EEA is reportedly around 25% and is continuously increasing (8). In Italy, foreign-born TB notifications raised from 39.4% in 2004 to 63% in 2013 (9-12). Higher TB rates have been reported in particular Italian regions due to immigration (13). In migrant populations, TB can be the outcome of the reactivation of an infection acquired in either the country of origin or in the host country, or during a visit to the country of origin. Native population might be even more contagious, following the diagnostic delay due to underestimation of the TB risk – by themselves or the health services – in people born in a low-risk area (14,15). In the UK, from 2008 to 2010, 20% recent immigrants from the Indian sub-continent and almost 30% from sub-Saharan Africa were LTBI carriers (16).

TB transmission in community settings – including schools – can be reduced through screening for early diagnosis of people living with LTBI, followed by rapid initiation of appropriate prophylactic therapy (17). Universal TB screening is contraindicated in low-incidence populations. However, the risk of contracting TB in households, communities, and congregate settings with shared living spaces and frequent social contacts is significantly higher (17-19). According to the US Centers for Disease Control and Prevention (CDC), LTBI screening should be conducted in high-risk individuals identified by a risk-assessment questionnaire (20). The CDC does not recommend TST or interferon gamma release assays (IGRA) for individuals without any known *Mycobacterium tuberculosis* exposure (21,22). Individuals at risk of exposure to TB according to CDC include (20):

• close contacts of a patient with active TB;

• migrants from high risk countries;

• individuals residing or working in communities or institutions with persons at high risk for TB, including TB clinics, reception centers for migrants, prisons, care homes, other.

An ideal school screening program should ensure that students and staff with LTBI or disease are immediately diagnosed and treated before attending school to avoid *Mycobacterium tuberculosis* transmission. The screening tests used for LTBI are the TST and IGRA (23), both only providing information on previous *Mycobacterium tuberculosis* exposure, based on the principle of cell mediated immunity (24).

In Japan, both new school teachers and students undergo TST followed by IGRA and an annual chest x-rays if appropriate (25). New York City requires a TB screening test (TST, IGRA, or chest x-ray) for all newly employed school teachers (26). In New Jersey (USA), TST or IGRA are required for all students born in high-incidence countries. The same TB screening protocol is compulsory also for all newly hired school employees (full-time as well as part-time), all school teachers, school bus drivers, and volunteers having a regular contact with students. TB testing is not required for:

• volunteers working with pupils for <20 hours per month;

• new employees, student teachers, and school contractors with a documented negative TB result (TST or IGRA) in the last six months or a documented positive TB test (TST or IGRA),

• school employees transferring between school districts or from a non-public school within New Jersey with a documented TB test result upon initial employment by a New Jersey school (27).

In Washington state (USA), all newly employed school staff need to show, within 12 months prior employment the following (28):

• a negative TST; or

• a negative IGRA; or

• a positive TST or a positive IGRA accompanied by: a negative chest x-ray or evidence of LTBI treatment.

Similar public health policies are also in place in other US states, such as South Carolina and Delaware, where school staff must provide the results of a test done within the previous 12 months during the first 15 working days of a new employment (29,30). School staff moving within Delaware need to show the latest TB test to the new district within 60 days of employment. Exemptions are granted only if this is in contrast with documented personal religious beliefs (30).

In California, a mandatory pre-employment clinical assessment was replaced by the Williams’ bill with a TB risk assessment questionnaire administered by a health care provider to all school staff (31,32). A similar approach is also in place in other Southern USA states such as Texas and Florida (21,33).

According to the British National Education Union, school teachers are not at high occupational risk of TB. This policy is supported by low TB rates in the general population, and by a high Bacillus Calmette–Guérin (BCG) vaccination coverage in the past (34). BCG vaccine was offered to secondary-school children until 2005 in the UK, when targeted programs for babies, children, and young adults at high risk of TB were implemented (35). Pre-employment health questionnaires include a general assessment for symptoms of pulmonary TB, which are needed for referral to further diagnostic workup (34). However, a pre-employment screening questionnaire to detect school staff potentially at high risk of TB who have close contact with students would probably not be sufficient for an effective TB prevention strategy in schools of low-incidence countries (31,32,36,37). Self-administered questionnaires screening seems more suitable to diagnose TB cases in high-risk populations (eg, reception centers for migrants) (38), but they are unable to effectively screen LTBI in low-risk communities (eg, schools).

It could be debated whether a TST-based screening could be more cost-effective than an IGRA-based screening or a dual-step screening with IGRA to confirm TST positive cases (25,39), although Portuguese data seem to suggest that IGRA may be a more effective strategy (40). It would also need to be clarified whether a different approach should be used for school teachers and students. On this note, 2016 guidelines of the UK National Institute for Health & Care Excellence recommend therapy of all infected children in case of a TST≥5 mm subsequently confirmed also by IGRA screening test (41). Treatment is however not required for children who are TST-positive but IGRA-negative (41). A multicenter study evaluating this policy showed that no children who were TST-positive but IGRA-negative and did not receive TB treatment developed incident TB over a two-year follow-up period (42).

## Conclusions

Effective TB screening policies should potentially be implemented in low-incidence countries where TB screening is not mandatory for school staff with direct contact with pupils. In Italy, although required by law, occupational health surveillance for school staff is in practice either neglected or limited to the use of video terminal and/or manual handling of loads, as there is an official consensus that “the majority of Italian schools do not present health risks to justify workplace health surveillance for their staff” (43-46). Possible school outbreaks as the one mentioned above cannot be curtailed without pro-active targeted screening for LTBI in school staff and children (16,47,48).
